# S16-2 National Physical Activity Strategies in Ireland

**DOI:** 10.1093/eurpub/ckae114.271

**Published:** 2024-09-26

**Authors:** Réka Veress

**Affiliations:** National School, University and Leisure Sport Federation, Hungary

## Abstract

**Purpose:**

The benefits of physical activity are well recognised across the world. They encapsulate individual physical and mental wellbeing and broader social, economic and environmental benefits to broader society. Ireland has adopted a cross-sectoral approaches to promoting physical activity, driven by the National Physical Activity Plan in 2016 and built on through the National Physical Activity Framework and Action Plan in Q2 2024. The objective of this intervention is to give the audience an outline of the factors which informed the development of national strategies, an overview of their governance and objectives, and where possible, an indication of the impacts such strategies have had on national physical activity levels.

**Policy or policy description:**

Ireland’s first National Physical Activity Plan was published in 2016, and represented the first joined-up approach across relevant sectors in the State, including Government, NGO, research and practitioner participation. It highlighted the range of benefits accruing from a physically active lifestyle and brought about a number of positive outcomes, chiefly in the areas of cross-sectoral collaboration and the targeting of specific population cohorts. Work on the development of its successor was commenced in 2023, which will be based on the approach of a framework to 2040 which will ensure coherence of approach across the relevant policyholders, and a series of action plans which will drive the achievement of its objectives.

Ireland is not alone in adopting such an approach to the coordination and promotion of physical activity initiatives. This session will inform participants from across the WHO European Region with similar approaches, to inform both policymakers and actors in the physical activity arena on how these linkages operate in Ireland at the HEPA Conference.

**Conclusion:**

The promotion of physical activity is of benefit to a large number of sectors, but a coherent approach requires buy-in and ongoing engagement across a range of policyholders. Developing a national strategy to promote physical activity requires a tailored approach, recognising particular challenges at national and regional level and across population cohorts. An outline by HEPA coordinators of the approaches taken and the learnings arising will be of significant interest to the HEPA audience.

